# Effect and mechanism of T lymphocytes on human induced pluripotent stem cell-derived cardiomyocytes via Proteomics

**DOI:** 10.1186/s13287-024-03791-4

**Published:** 2024-07-29

**Authors:** Jin Ye, Sichi Xu, Xiaoqing Liu, Qiyu Zhang, Xiao Li, Hui Zhang, Jie Ma, Ling Leng, Shuyang Zhang

**Affiliations:** 1 Stem cell and Regenerative Medicine Lab, Institute of Clinical Medicine, State Key Laboratory of Complex Severe and Rare Diseases, Translational Medicine Center, Peking Union Medical College Hospital, Chinese Academy of Medical Sciences and Peking Union Medical College, Beijing, 100730 China; 2 Department of Cardiology, Peking Union Medical College Hospital, Chinese Academy of Medical Sciences and Peking Union Medical College, Beijing, 100730 China; 3 State Key Laboratory of Medical Proteomics, Beijing Proteome Research Center, National Center for Protein Sciences (Beijing), Beijing Institute of Lifeomics, Beijing, 102206 China; 4 College of Life Sciences, Hebei University, Baoding, 071002 China; 5 Department of Pathology, Peking Union Medical College Hospital, Chinese Academy of Medical Sciences and Peking Union Medical College, Beijing, 100730 China

**Keywords:** hiPSC-derived cardiomyocytes, Myocarditis, Proteomics, T lymphocytes

## Abstract

**Background:**

Abnormalities in T cell activation play an important role in the pathogenesis of myocarditis, and persistent T cell responses can lead to autoimmunity and chronic cardiac inflammation, as well as even dilated cardiomyopathy. Although previous work has examined the role of T cells in myocarditis in animal models, the specific mechanism for human cardiomyocytes has not been investigated.

**Methods:**

In this study, we constructed the human induced pluripotent stem cell-derived cardiomyocytes (hiPSC-CMs) and established the T cell-mediated cardiac injury model by co-culturing with activated CD4 + T or CD8 + T cells that were isolated from peripheral mononuclear blood to elucidate the pathogenesis of myocardial cell injury caused by inflammation.

**Results:**

By combination of quantitative proteomics with tissue and cell immunofluorescence examination, we established a proteome profile of inflammatory myocardia from hiPSC-CMs with obvious cardiomyocyte injury and increased levels of lactate dehydrogenase content, creatine kinase isoenzyme MB and cardiac troponin. A series of molecular dysfunctions of hiPSC-CMs was observed and indicated that CD4 + cells could produce direct cardiomyocyte injury by activating the NOD-like receptor signals pathway.

**Conclusions:**

The data presented in our study established a proteome map of inflammatory myocardial based on hiPSC-CMs injury model. These results can provide guidance in the discovery of improved clinical treatments for myocarditis.

**Supplementary Information:**

The online version contains supplementary material available at 10.1186/s13287-024-03791-4.

## Background

Myocarditis and inflammatory cardiomyopathy are categorized as part of a spectrum of heart-specific inflammatory responses, which present with distinct clinical and histopathological features [1, 2]. Prolonged activation of the inflammatory response can result in structural and functional impairments, leading to the development of dilated cardiomyopathy (DCM) [3]. Previous research has indicated that as much as 40% of DCM cases are linked to inflammation or viral infection. Additionally, myocarditis has been identified as a cause of sudden cardiovascular death, accounting for almost 2%, 5%, and 5–12% of sudden deaths in infants, children, and young athletes, respectively [4]. It has been reported that the prevalence of myocarditis ranges from 10.2 to 105.6 per 100,000 worldwide [5]. Inflammatory signals control immune cells, especially T cells, which are implicated in the genesis and progression of myocarditis and inflammatory cardiomyopathy. Although T cells serve as a critical factor in myocarditis development, they only constitute a minority of heart-infiltrating cells in both mouse models of myocarditis and in human myocarditis cases subjected to biopsy [6]. T-cell-mediated immune responses are intertwined with a diverse spectrum of cardiac conditions, encompassing traditional inflammatory disorders like myocarditis and post-myocardial infarction syndrome, as well as non-inflammatory conditions such as hypertensive and diabetic cardiomyopathy [7]. Moreover, CD4 + T cell responses, specifically, have the capacity to transform an initial myocarditis condition into an autoimmune reaction [8, 9]. Robust evidence supports the pivotal role of T lymphocytes in the pathogenesis of cardiac diseases through their ability to amplify the inflammatory functions of surrounding cells [10].

Despite considerable advancements in exploring the consequences of pathog enic T cell responses on heart diseases, the absence of an in-depth understanding of the particular mechanisms that T cells modulate during the early stages of these diseases has hindered the development of efficacious and secure therapeutic interventions that aim to target the immune system in chronic inflammatory cardiac conditions. Although previous study has identified some regulatory pathways of myocarditis in a mouse myocarditis model, we still lack sufficient evidence and specific myocardial cytotoxicity of T cell on human cardiomyocytes [11]. Introduced in 2007 by both Dr. Shinya Yamanaka and the Thomson group, the concept of human induced pluripotent stem cells (hiPSCs) encompasses cells that offer numerous advantages. These include boundless proliferation, the potential to differentiate into any cell type within the human body, and the ability to generate patient-specific isogenic cells for disease modeling [12, 13]. Through the utilization of hiPSCs, the generation of cardiac-specific cells from both healthy donors and patients has been made possible. This process involves the reprogramming of somatic cells obtained via skin biopsies or blood draws into hiPSCs, which can subsequently be differentiated into cardiac cells. Moreover, this technology has largely eliminated the restriction of diversity of species. Thus, hiPSC-CMs have been widely used as in vitro models in arrhythmias, hereditary cardiomyopathy, and drug-related cardiotoxicity researches [14–16]. The specific influence of inflammatory cells and T cells on hiPSC-CMs has not yet been discussed. In this study, we adopted co-culturing of hiPSC-CMs and different typed of T cells to elucidate the specific mechanisms of T cell-mediated cardiac injury.

## Methods

### Human samples

The paraffin-embedded heart tissue samples were acquired from the Department of Pathology, Peking Union Medical College Hospital. All tissue and blood samples, as well as clinical data, were collected with the informed consent of the patients’ guardians, and the procedures were conducted in compliance with the regulations of the hospital ethics committee, the National Health Commission of China, and the Helsinki Declaration. Ethical approval for this study was obtained from the Peking Union Medical College Hospital (JS-2901).

### Generation of hiPSC-CMs

The hiPSC was obtained from Nuwacell Biotechnologies Co., Ltd (RC01001-B; Female). The generation of hiPSC-CMs was performed in the way described in a previous study [17]. Briefly, the pluripotency of hiPSCs was first verified using immunofluorescence staining of OCT4 (PA5-27438, invitrogen) and SOX2 (561,469, BD Biosciences). The hiPSCs were first cultured as small cell clusters in TeSR-E8 medium (Stemcell 05990, STEMCELL Technologies) containing 10 µM ROCK inhibitor (Y27632, Selleck Chemicals) on a six-well plate coated with Matrigel (Corning 354,277, Corning Incorporated), for four days prior to differentiation. The culture medium was replaced daily with TeSR-E8 medium without ROCK inhibitor. Cardiac differentiation was then induced using a STEMdiff Cardiomyocyte Differentiation Kit (05010, STEMCELL Technologies), following the manufacturer’s instructions. The cardiomyocytes were identified using immunofluorescence staining of actinin (AB7811, Sigma-Aldrich) and cardiac troponin T (cTnT) (ab45932, Abcam).

### Isolation of peripheral blood mononuclear cells and flow cytometric analysis

Healthy volunteers provided human peripheral blood, from which the fresh venous blood was collected to isolate peripheral blood mononuclear cells (PBMCs).The isolation of PBMCs was performed using density-gradient centrifugation with Lymphoprep (P8610, Solarbio, China) to separate the cells from the buffy coat. After isolation, PBMCs were washed in PBS buffer supplemented with 0.5% BSA and 2 mM EDTA. Following this step, the cells were incubated with a cocktail of antibodies, which included PE/Cyanine7-conjugated anti-human CD3 antibody (317,334, BioLegend), FITC-conjugated anti-human CD4 antibody (317,408, BioLegend), and APC-conjugated anti-human CD8 antibody (344,722, BioLegend) for 30 min at 4 °C. Then the CD3 + and CD4 + T cells and CD3 + and CD8 + T cells were analyzed and sorted by the flow cytometry using either a BD LSRII flow cytometer or a BD LSRFortessa SORP following the standard protocols.

### Co-culture and enzyme-linked immunosorbent assay (ELISA) analysis

The CD3 + and CD4 + T cells and CD3 + and CD8 + T cells obtained from flow cytometric analysis were resuspended in a complete medium composed of RPMI supplemented with 10% heat-inactivated fetal bovine serum, 1% L-glutamine, and 1% penicillin-streptomycin. After that, these T cells underwent activation through the stimulation of Dynabeads human CD3/CD28 (11132D, Thermo-Fisher Scientific) and recombinant human IL-2 (PHC0021, Thermo-Fisher Scientific) at the prescribed beads-to-cell ratio of 1:1. Then, hiPSC-CMs were introduced at a 1:10 proportion to the activated CD4 + and CD8 + T cells (1 × 10^6^) for direct co-cultures, establishing a physical connection, and subsequently incubated at 37 °C under a 5% CO2 atmosphere for a duration of 48 h [18]. The co-culture was sustained for 48 h, and the supernatant of the basal media of hiPSC-CMs was collected precisely 48 h after co-culture initiation and promptly preserved at − 80 °C. Meanwhile, the supernatant of the basal media of hiPSC-CMs co-cultured without T cells was collected as controls. The levels of lactate dehydrogenase (LDH), creatine kinase isoenzyme MB (CK-MB), and cardiac troponin I (cTnI) in the supernatant of hiPSC-CMs co-cultured with and without the activated CD4 + and CD8 + T cells were quantified by employing ELISA kits, including an LDH kit (ml024518, Shanghai Enzyme linked Biotechnology Co., Ltd, China), a CK-MB kit (ml026270, Shanghai Enzyme linked Biotechnology Co., Ltd, China), and a cTnI kit (ml058644, Shanghai Enzyme linked Biotechnology Co., Ltd, China).

### RNA isolation and real-time PCR reaction

Total RNA was extracted utilizing the RNeasy® UCP Micro Kit (Qiagen, Germany, cat. no. 73,934) following the manufacturer’s prescribed guidelines. Subsequently, cDNA synthesis was achieved through reverse transcription RT-PCR (TaKaRa, Japan, RR036A, RR820A), and real-time PCR employing SYBR green master mix was conducted on a Bio-Rad iQ5 Real-Time PCR detection system. Acquisition of data was executed using Bio-Rad CFX Manager software, and the expression levels of designated genes within each sample were normalized against GAPDH expression utilizing the 2 − ΔΔCt method. The specific primer sequences for PCR are outlined in Table S1.

### Histological analysis

After fixing the cells in 4% formaldehyde for 20 min, washing them with PBS helps to remove any excess fixative and prepare the cells for subsequent staining steps. This step is important to ensure accurate and specific immunofluorescence labeling of the target proteins or molecules of interest. The treated cells were then blocked in 10% goat serum for 1 h at room temperatureand incubated with primary antibodies overnight at 4 °C. Following this, one-hour incubation of the cells with secondary antibodies at room temperature ensued, followed by a counterstaining procedure utilizing DAPI. The specimens were then hermetically sealed with Fluoro-Gel to facilitate the photography process. Negative control samples underwent exclusive incubation solely with secondary antibodies. Images were captured at magnifications of 20×/40× and subsequently subjected to meticulous analysis employing Volocity Demo (×64). As for the biopsied heart tissue, it was swiftly excised, that specimens were immobilized in a solution containing 4% paraformaldehyde, subsequently embedded within paraffin material, and then sliced into sections with a thickness of 4 μm. The tissue sections were permeabilized with 0.2% Triton X-100 for 20 min and blocked with 10% normal goat serum for 60 min at RT. Ventricular sections were incubated with primary antibodies against α-actinin (AB7811, Sigma-Aldrich), cTnT antibody (45,932, Abcam), IL-18 (10663-1-AP proteintech), ASC (SAB4501315, Sigma-Aldrich), NLRP-3 (NBP2-12446 Novus Biologicals), and subsequently with fluorescent secondary antibodies. Multiple sections of each heart, with a thickness ranging from 4 to 5 mm, were meticulously prepared and subjected to staining using hematoxylin and eosin (H&E) in order to assess histopathological features. In order to conduct immunohistochemical analysis, the heart sections were subjected to antigen retrieval utilizing the pressure cooker method. Subsequently, the sections were incubated with anti-CD3 (abcam, AB699), CD4 (19068-1-AP, proteintech), CD8 (66868-1-Ig, proteintech), and CD68 (Thermo-Fisher 66231-2-Ig) antibodies. The specimens were then treated with goat anti-rabbit EnVisionTM +/HRP reagent, and stained using a DAB detection kit.

### Protein extraction, digestion, and mass spectrometry (MS) analysis

To initiate the sample preparation, a 100 µg aliquot of cells was subjected to precipitation with four-fold volumes of frigid acetone. The mixture was thoroughly blended, and left to precipitate at sub-zero temperatures for a period of one night. Following this, centrifugation was conducted at 14,000 g for a duration of 10 min, after which, a single milliliter of cold acetone was included to facilitate washing and precipitation. The sample was then dessicated under ambient conditions, before 100 µL of 8 M UA was utilized to accomplish re-dissolution. The resultant admixture was subjected to centrifugation at 14,000 g for a period of 20 min, from which the supernatant was harvested and fractionated. Subsequently, a 100 µg aliquot of extracted proteins was treated with 200 mM DTT solution to effect reduction, whilst incubated for duration of 1 h at an optimum temperature of 37 °C. Dilution was achieved by subjecting the sample to a four-fold decrease in concentration via a 25 mM ammonium bicarbonate buffer. In addition, trypsin was added to establish a trypsin-to-protein ratio of 1:50, followed by a subsequent overnight incubation at a temperature of 37 °C. The digestion reaction was then arrested by adding 50 µL of 0.1% FA on the ensuing day.

To construct comprehensive spectral libraries, we performed shotgun proteomics analyses utilizing the cutting-edge EASY-nLC 1200 ultra-high pressure system, coupled with the advanced Q Exactive HF-X mass spectrometer manufactured by Thermo-Fisher Scientific. The mass spectrometer was operated in a highly efficient data-dependent acquisition (DDA) mode. Specifically, the instrument employed the Top-40 data-dependent approach, wherein MS spectra were meticulously captured using the Orbitrap mass analyzer. These MS spectra were obtained at an impressive resolution of 120,000, covering a mass-to-charge ratio (m/z) range of 350–1500. To ensure optimal data quality, automatic gain control (AGC) was employed, targeting a value of 3 × 10^6^. Additionally, a maximum ion injection time of 80 ms was set to extract as much information as possible.

The most prominent ions from the full scan were selectively isolated using a narrow isolation width of 1.6 m/z. To generate tandem mass spectra, higher-energy collisional dissociation was applied with a normalized collision energy (NCE) value of 27. The resulting MS/MS spectra were obtained using the Orbitrap at a resolution of 15,000. For these spectra, an AGC target of 5 × 10^4^ was employed, along with a maximum ion injection time of 45 ms to capture sufficient signal intensity.

To enhance data quality and minimize redundancy, a precursor dynamic exclusion window of 16 s was enabled. This safeguarded against repeated selection of the same precursor ion within this time frame, thus maximizing the diversity of identified peptides. Overall, this state-of-the-art setup and methodological approach ensured the acquisition of high-quality spectral data, enabling accurate and comprehensive construction of spectral libraries for subsequent analysis.

The cellular specimens underwent analysis utilizing the data-independent acquisition (DIA) scanning mode. The MS1 resolution, positioned at 200 m/z, was configured to 60,000, while the MS2 resolution was set to 30,000. Within the m/z range of 350 to 1,500, the acquisition process was partitioned into 42 discrete windows, with isolation windows spanning from 14 to 312 m/z. The full scan AGC target was established at 3 × 10^6^, accompanied by an injection time of 80 ms. Regarding the DIA parameters, NCE values of 25.5%, 27%, and 30% were implemented, alongside a target value of 1 × 10^6^. To allow for uninterrupted operation of the MS in simultaneous ion replenishment and detection mode, the maximum injection time was configured to automatic.

The hybrid library creation process involved the utilization of MS data from fractionated pools (DDA MS data, 3 fractions) and individual subject samples (DIA MS data). This was achieved through the employment of Spectronaut software from Biognosys, specifically version 15.7.220308.50606. The resulting hybrid spectral library served as the basis for conducting protein identification and quantitation analyses on the MS data derived from the single-shot samples using the Spectronaut software. During the searches, the human UniProt reference proteome was employed as the search database, encompassing canonical and isoform sequences amounting to 75,093 entries, which were downloaded in August 2020. Fixed modification parameters were set to carbamidomethylation, while variable modifications included acetylation of the protein N-terminus and oxidation of methionine. Default settings were applied for other parameters.Enzymatic digestion was performed using the trypsin/P proteolytic cleavage rule, allowing for a maximum of two missed cleavages and considering peptide lengths ranging from 7 to 52 amino acids. To ensure accurate protein quantitation, the local normalization algorithm within Spectronaut, which relies on a local regression model, was utilized to normalize protein intensities.The generation of the spectral library required a minimum of three fragments per peptide, with a maximum inclusion limit of the six best fragments. A false discovery rate (FDR) of 1% was employed for both protein and precursor identification, and protein quantities were only reported for samples if the corresponding proteins passed the filter.

### Statistical and bioinformatics analysis

For the quantification values obtained for the identified proteins, a normalization process was performed. This involved taking a fraction of the total quantification value, followed by multiplying it by 10^6^ to scale it up. Subsequently, a log2 transformation was applied to the scaled quantification values. This normalization and transformation step helps in standardizing the protein quantification values and allows for easier interpretation and comparison of protein expression levels across different samples. Pairwise comparison was performed by a moderated t-statistic using the R package Limma (version 3.50.0) to identify proteins whose expressions differed significantly between CD4+/CD8 + T cell-treated cardiomyocytes and control cardiomyocytes. Benjamini-Hochberg (BH) adjusted p-values < 0.01 were considered statistically significant. Proteins that met the fold-change threshold were categorized as upregulated (log2 CD4+/CD8 + T cell-treated/control > 1) or downregulated (log2 CD4+/CD8 + T cell-treated/control < -1). Experimental data were presented as means ± standard deviations, and statistical comparisons between groups were conducted using Student’s t-test for two-group comparisons and ANOVA for three or more group comparisons. Each experiment was conducted a minimum of three times (*n* ≥ 3). Statistical significance was determined at *p* ≤ 0.05 (*), 0.01 (**), or 0.001 (***) levels. GraphPad Prism (version 9.0.0) was used to perform all statistical tests.

## Results

### Differentiation of hiPSC-CMs

**Fig. 1:**
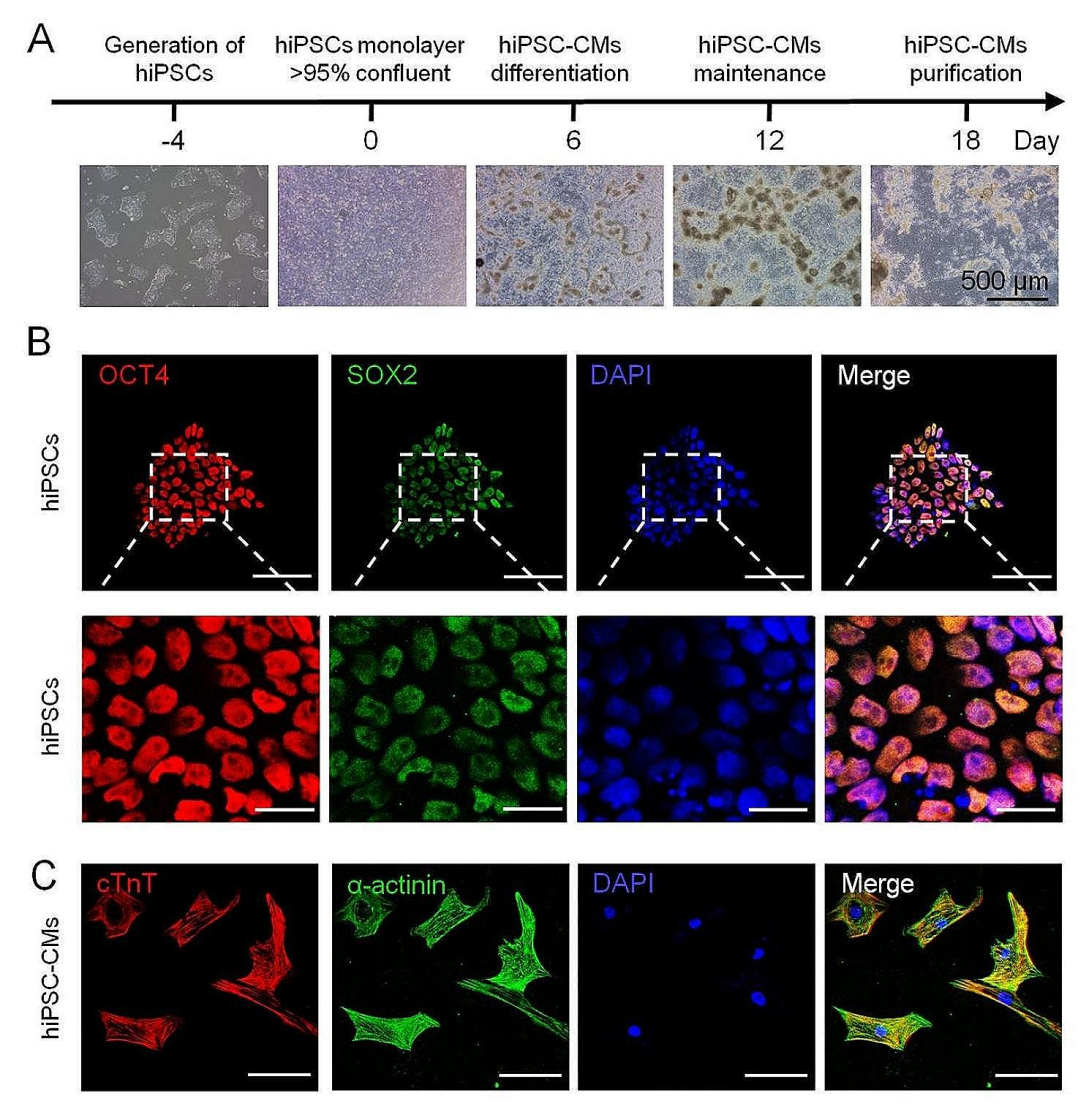
Overview of differentiation and identification of iPSC-CMs. (**A**) Schematic of human induced pluripotent stem cell-derived cardiomyocytes (hiPSC-CMs) differentiation and culture. The light areas represent hiPSC-CMs cultures on days 0, 6, 12, and 18 (scale bar: 500 μm). (**B**) Immunofluorescence of typical hiPSC markers OCT4 (red) and SOX2 (green) (scale bar: 100 μm). (**C**) Representative expression of mature cardiomyocyte-specific biomarkers of cardiac troponin T (cTnT) (red) and α-actinin (green) (scale bar: 100 μm)

To investigate the specific mechanisms of inflammation on human cardiomyocytes, we induced and established hiPSC-CMs in vitro, as previously described [17]. The primary differentiation process of the cardiomyocytes is depicted in Fig. 1A. The stable hiPSC lines were subcultured and cryopreserved for over 15 generations. Subsequently, the hiPSCs were stained for pluripotency markers (OCT4 and SOX2) and detected using immunofluorescence techniques (Fig. 1B). Before differentiation begun on day 0, the high-quality hiPSCs should reach above 95% confluency with a density of 3.5–8 × 10^5^ cells/well. After 6 days of differentiation, clusters of cardiomyocytes had been formed, and small areas of beating cardiomyocytes were visible (Fig. 1A). We used cardiomyocyte maintenance medium for another 6 days to generate mature cardiomyocytes and to improve the maturity of the cardiomyocytes. The mature cardiomyocytes were processed for another 6 days with specific cardiomyocyte-purified medium to improve cardiomyocyte purity. Then, the successfully differentiated hiPSC-CMs were identified using the specific marker of cTnT and α-actinin (Fig. 1C). By analysis of flow cytometry method, the purity of hiPSC-CMs is about 91.5% (Figure S1A) and the calcium transient recordings of beating clusters of cardiomyocytes were observed (Figure S1B).

### Proteome profile of hiPSC-CMs with inflammatory T cells infiltration

**Fig. 2:**
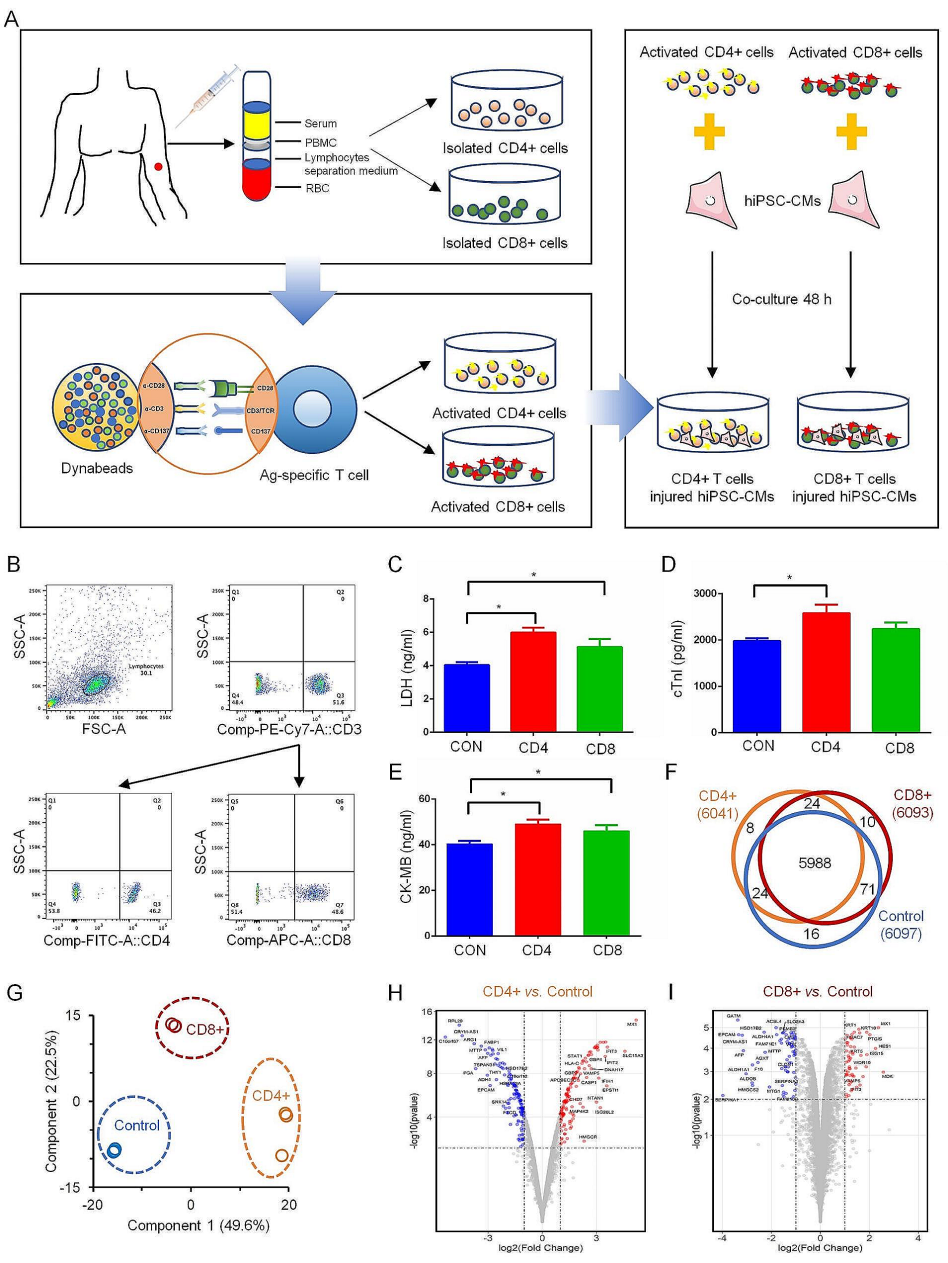
Proteome profile of injured hiPSC-CMs cultured with activated T cells. (**A**) Overview of study designs from blood collection, peripheral blood mononuclear lymphocytes (PBMC) isolation, T cell activation, and co-culture with hiPSC-CMs. (**B**) Isolation of CD4 + and CD8 + T cells from PBMC with flow cytometry. (**C**–**E**) Expression level of cardiomyocyte injury markers of lactate dehydrogenase (LDH), creatine kinase-MB (CK-MB), and cardiac troponin I (cTnI), detected by ELISA. * *p* < 0.05. (**F**) Venn diagram represents the number of overlapping proteins identified among hiPSC-CMs (Control), CD4 + T cell-treatment hiPSC-CMs (CD4+), and CD8 + T cell-treatment hiPSC-CMs (CD8+). (**G**) Principal coordinates analysis (PCoA) of the proteome profile in hiPSC-CMs treated by CD4 + and CD8+, as well as normal hiPSC-CMs. (H, I) Volcano plots of -log10 p-values vs. log2 protein abundance comparisons between CD4 + and CD8 + T cell-treated hiPSC-CMs and normal controls

To investigate the direct effects of T cells on hiPSC-CMs, we adopted a density-gradient centrifugation with human peripheral blood lymphocyte separation solution to acquire PBMCs from healthy donors (Fig. 2A). As shown in Fig. 2B, CD3 + and CD4 + T cells and CD3 + and CD8 + T cells were successfully divided and obtained. Following previous studies [17, 18], the T cells were activated using anti-CD3/CD28 dynabeads in combination with IL-2 as a mitogenic stimulus. Subsequently, the activated T cells were co-cultured with hiPSC-CMs for 48 h, and the cell supernatant was collected to detect markers associated with cardiac injury. As depicted in Fig. 2C–E, CD3 + and CD4 + T cells, as well as CD3 + and CD8 + T cells, exhibited a significant increase expression of LDH, CK-MB, and cTnI, suggesting the presence of stress effects induced by T cells. Also, the calcium transient recordings showed the beat rates of hiPSC-CMs decreased after co-cultured with the CD4 + and CD8 + T cells (Figure S1C, D).

Subsequently, a quantitative proteomic analysis was conducted to investigate the pathological characteristics of the injured cardiomyocytes. The results revealed a wide range of protein intensities spanning across seven orders of magnitude (Figure S2A). Additionally, strong correlations were observed between the duplicate datasets within each group (Figure S2B). In total, 6138 proteins were identified in all cardiomyocytes samples, comprising 6096 proteins in normal cardiomyocytes, 6041 proteins in CD4 + T cell-injured cardiomyocytes, and 6092 proteins in CD8 + T cell-injured cardiomyocytes (Table S2). Principal coordinates analysis (PCoA) revealed that the proteins identified in the injured cardiomyocytes and those present in the control tissue formed distinct clusters (Fig. 2G). Further analysis identified a total of 243 and 120 proteins as differentially expressed (statistical test: BH adjusted *p* < 0.01) in the injured cardiomyocytes with CD3 + and CD4 + or CD3 + and CD8 + T cell treatments, respectively, when compared to the control (Table S3, S4). Among the identified proteins, 122 were upregulated (> 2-fold), and 121 were downregulated (< 2-fold) in response to CD4 + T cell treatment (Fig. 2H), while 54 were upregulated (> 2-fold), and 66 were downregulated (< 2-fold) in response to CD8 + T cell treatment (Fig. 2I).

### Inflammatory T cell infiltration leads to dysfunction of hiPSC-CMs

**Fig. 3:**
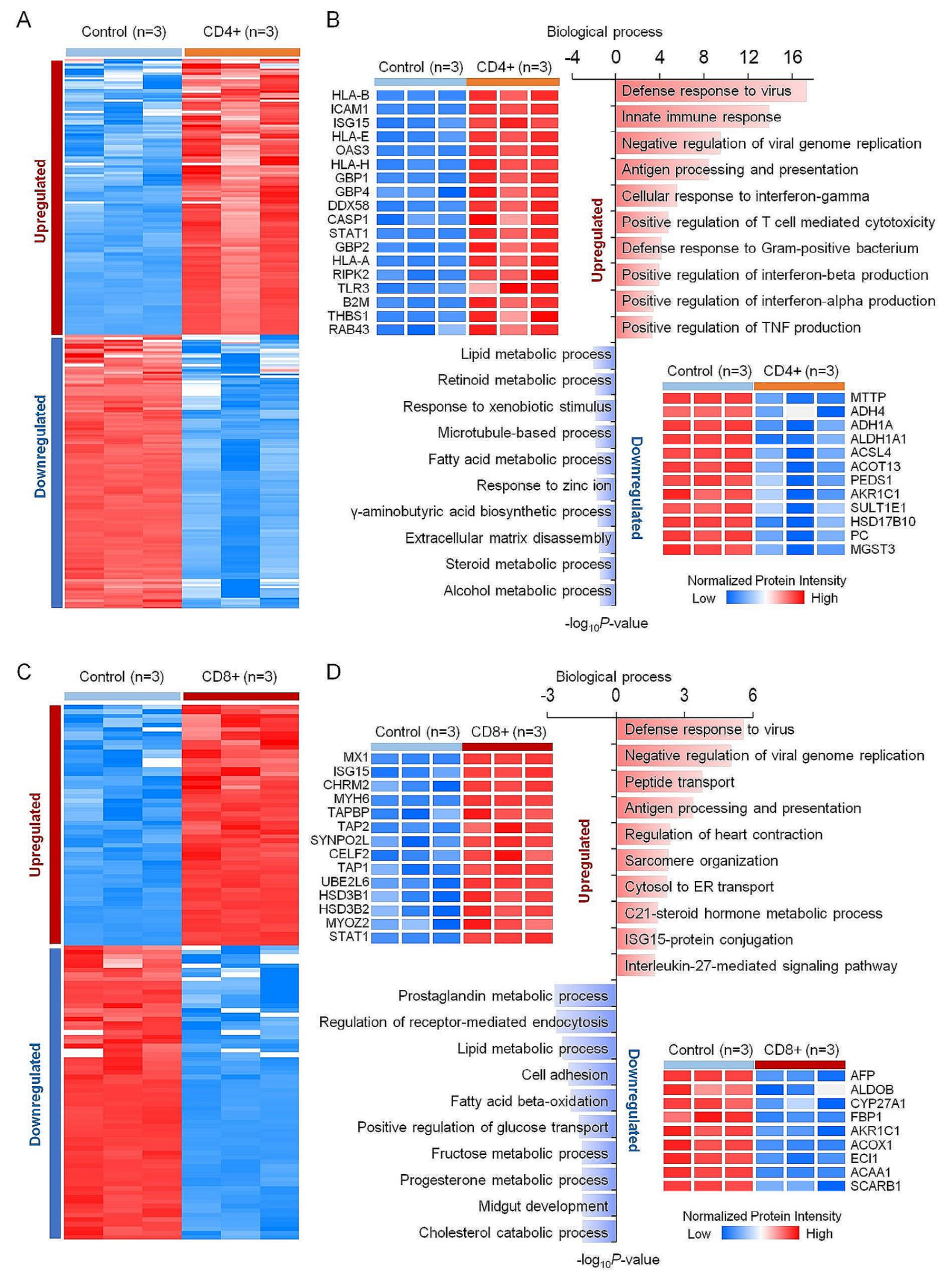
Functional analysis of differentially expressed proteins of injured hiPSC-CMs, cultured with activated T cells. Heatmaps of differentially expressed proteins (DEPs) between CD4+ (**A**) or CD8+ (**B**) cell-treated hiPSC-CMs and control groups. The red and blue boxes indicate proteins with increased and decreased abundance, respectively. Biological processes of DEPs in CD4+ (**C**) and CD8+ (**D**) cell-treated hiPSC-CMs are grouped according to the -log10 p value of the degree of enrichment. The DEPs were determined based on the moderated t-statistic using the R package Limma with the Benjamini–Hochberg (BH) adjusted *p* < 0.01 and log2 CD4+/Control > 1 or log2 CD8+/Control > 1 (upregulated), and BH adjusted *p* < 0.01 and log2 CD4+/Control < − 1 or log2 CD8+/Control < − 1 (downregulated)

The upregulated proteins in the CD4 + T cell treated hiPSC-CMs compared to the normal control group, were enriched in response to microorganisms, including a defensive response to a virus and a Gram-positive bacterium. Also, several immune response related proteins including the interferon-gamma (RAB43, STAT1, CASP1, GBP2, GBP1, GBP4, TLR3, and ICAM1), interferon-beta (DDX58, RIPK2, OAS3, ISG15, and TLR3), interferon-alpha production (DDX58, STAT1, RIPK2, and TLR3), tumor necrosis factor production (DDX58, RIPK2, OAS3, THBS1, TLR3, and HLA-E), and T cell-mediated cytotoxicity (HLA-H, HLA-B, HLA-A, B2M, and HLA-E) associated proteins were highly expressed in the CD4 + T cell treatment group, indicating that CD4 + T cells that infiltrate myocardial cells are activated and secrete a variety of inflammatory factors to resist microorganisms (Fig. 3A, B). However, proteins associated with metabolic processes, including lipid metabolic process (PC, MGST3, MTTP, ACOT13, ACSL4, and HSD17B10), retinoid metabolic process (ADH4, AKR1C1, and ALDH1A1), fatty acid metabolic process (MSMO1, PEDS1, ACSL4, and HSD17B10), steroid metabolic process (SULT1E1, AKR1C1, and MSMO1), and alcohol metabolic process (ADH4 and ADH1A) had low expression in the CD4 + T cell treatment group, compared to the normal control group (Fig. 3A, B). In addition, proteins associated with microtubule-based and extracellular matrix disassembly processes also had low expression in the CD4 + T cell treatment group. We found the same results in the CD4 + T cell treatment group, such that several upregulated proteins were enriched in the defensive responses to viruses in the CD8 + T cell treatment (Fig. 3C, D). By contrast, we found most upregulated proteins in the CD8 + T cell treatment group were enriched in peptide transport (TAP2, TAP1, and TAPBP), heart contraction (CHRM2, CELF2, and MYH6), sarcomere organization (SYNPO2L, MYOZ2, and MYH6), C21-steroid hormone metabolic process (HSD3B2 and HSD3B1), ISG15-protein conjugation (ISG15 and UBE2L6), and Interleukin-27-mediated signaling pathway (STAT1 and MX1). The results also showed that proteins associated with metabolic processes, including fatty acid beta-oxidation (ACOX1, ECI1, and ACAA1), fructose metabolic process (ALDOB and FBP1), progesterone metabolic process (AKR1C1 and AFP), cholesterol catabolic process (CYP27A1 and SCARB1), which was not found in the low expressed proteins of the CD4 + T cell treatment group, were low expressed in the CD8 + T cell treatment group (Fig. 3C, D). These results indicated that CD4 + and CD8 + T cell treatment have something in common with respect to the biological process of cardiomyocyte injury, such as in increased immune response and decreased metabolism level. However, CD4 + T cell treatment tends to produce interferons and tumor necrosis factor, which destroys retinal, fatty acid, and cholesterol metabolism of cardiomyocyte, as well as the related cytoskeleton and tissue skeleton of microtubules and extracellular matrix. CD8 + T cell treatment tend to affect cardiac contraction and glucose metabolism of cardiac function.

### Activation of the nucleotide oligomerization domain NOD-like receptor pathway leads to cardiomyocyte inflammation

**Fig. 4:**
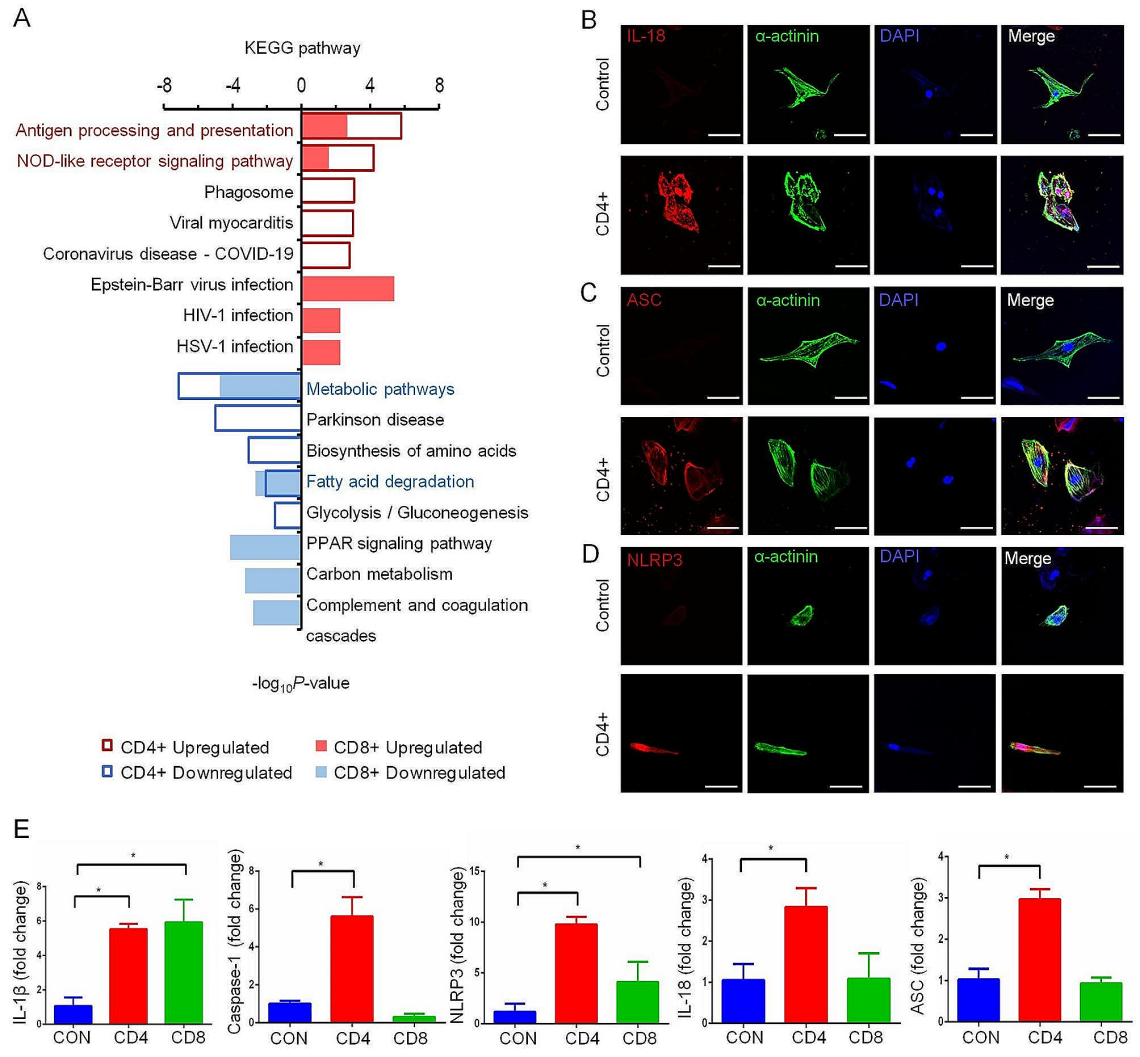
NLR signal pathway activated in injured hiPSC-CMs cultured with activated T cells. (**A**) Functional analyses of upregulated and downregulated DEPs identified in the CD4 + and CD8 + T cell-treated hiPSC-CMs compared with controls. The DEPs were determined based on the moderated t-statistic using the R package Limma with the BH adjusted *p* < 0.01 and log2 CD4+/Control > 1 or log2 CD8+/Control > 1 (upregulated), or BH adjusted *p* < 0.01 and log2 CD4+/Control < − 1 or log2 CD8+/Control < − 1 (downregulated). (**B**–**D**) Immunofluorescence of the primary NOD-like receptor pathways, such as IL-18, ASC, and NLRP3 in CD4 + T cell-treated hiPSC-CMs and control group (scale bar: 100 μm). (**E**) Transcription level of IL-1β, caspase-1, NLRP3, IL-18, and ASC in CD4 + and CD8 + T cell-treated hiPSC-CMs and control groups

In order to explore the inflammatory activation pathway of T cells acting on hiPSC-CMs, we conducted a KEGG pathway analysis. The results revealed that the metabolic pathway exhibited a diminished expression in both the CD4 + T cell and CD8 + T cell treatment hiPSC-CMs (Fig. 4A). In particular, the treatment with CD4 + T cells primarily affected the synthesis of amino acids, while the treatment with CD8 + T cells resulted in the downregulation of PPAR and carbon metabolism. Interestingly, both CD4 + T cell and CD8 + T cell treatments led to the upregulation of antigen processing and presentation, as well as the NOD-like (NLR) pathway (Fig. 4A). Previous studies have established that categorization of the NLR protein family as constituents of a cluster of pattern recognition receptors (PRRs) accountable for instigating the innate immune response to cellular harm and strain. The inflammasomes, comprising an NLR, the adapter protein ASC, and the operative entity pro-caspase-1 [19], assume a pivotal function in this intricate progression. When activated, inflammasomes facilitate the cleavage and initiation of caspase-1, resulting in the liberation of pro-inflammatory cytokines IL-1β and IL-18. To further investigate the activation of the NLR pathways, we evaluated the protein concentrations of NLRP3, ASC, and IL-18 of NOD-like receptor pathways within the cardiomyocytes by means of immunofluorescence (as portrayed in Fig. 4B–D). The results showed that just similar as the proteomic analysis data, co-culturing with CD4 + T cells can lead to obviously increased levels of IL-18, ASC, and NLRP3 in the hiPSC-CMs, compared with the normal group. At the same time, we found that both CD4 + T cells and CD8 + T cells had elevated mRNA levels of IL-1β, caspase-1, NLRP3, IL18, and ASC of hiPSC-CMs (Fig. 4E).

**Fig. 5:**
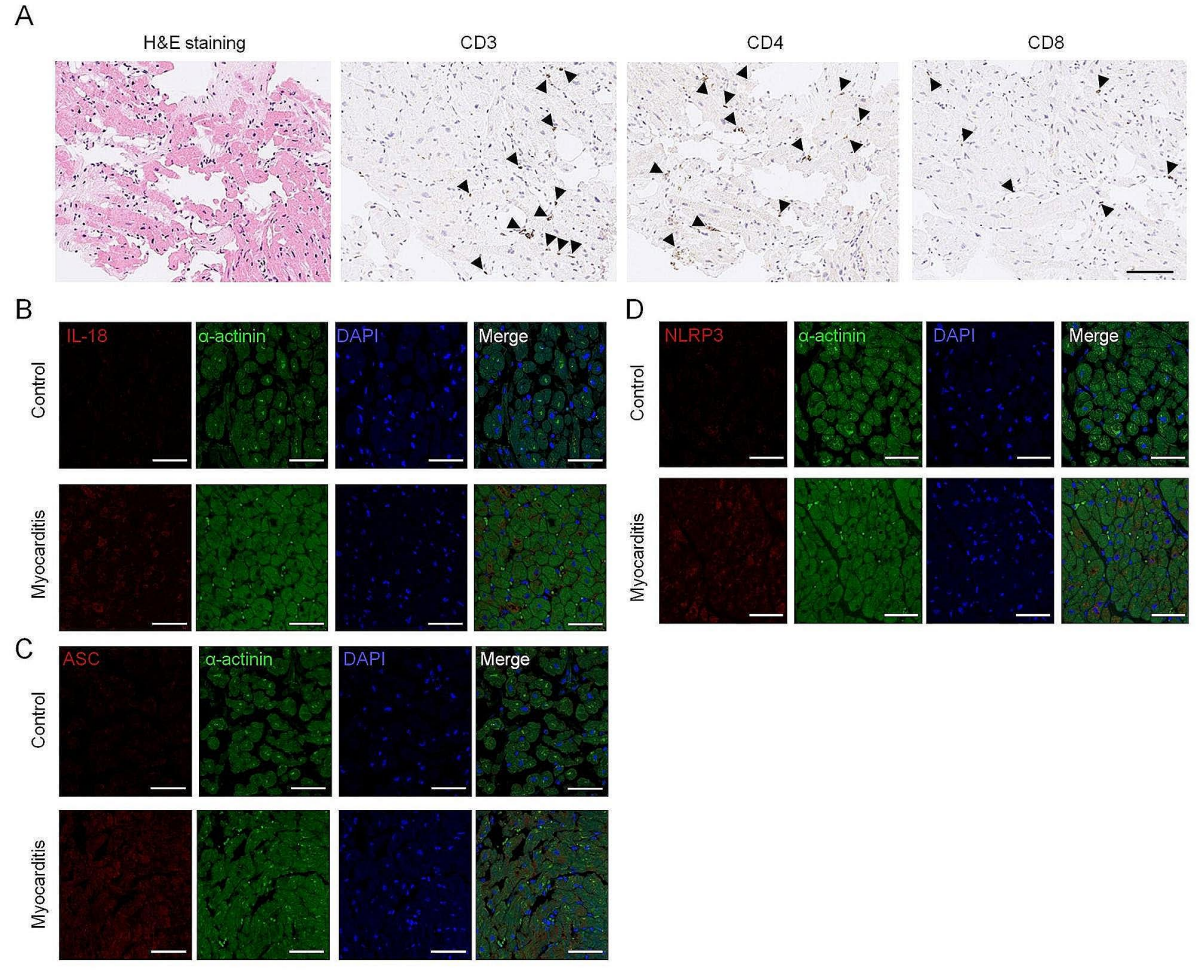
NLR signal pathway activated in heart tissues with myocarditis. (**A**) Hematoxylin and eosin (H&E) and immunohistochemistry stainings of CD3, CD4, and CD8 in heart tissues with myocarditis (scale bar: 100 μm). The black arrows refer to the T cells around the myocardium or cardiovascular of the heart tissues with myocarditis. (**B**–**D**) Immunofluorescence of the IL-18, NLRP3, and ASC in the heart tissues with myocarditis and non-myocarditis (control) groups (scale bar: 100 μm)

Furthermore, there is currently no report on the specific role of NLR pathways in human cardiac tissues. To validate our findings, we conducted tests to examine the expression of NLR in patients diagnosed with myocarditis. The patients were diagnosed with myocarditis with scattered lymphocyte infiltration, especially for CD4 + cells in the myocardium (Fig. 5A). As depicted in Fig. 5B–D, the expression of NLR was notably activated, as evidenced by elevated levels of ASC, IL-18, and NLRP3 in human cardiac tissues. It is interesting to note that the observations in the hiPSC-CMs treated with T cells align with the findings regarding the beneficial effects of corticosteroids on cardiac function in viral myocarditis patients. This suggests a potential translational relevance between in vitro models and clinical outcomes.

## Discussion

Myocarditis is a condition characterized by inflammation process on the muscular tissue of the heart. It is a significant contributor to sudden death, as evidenced by autopsy-based investigations carried out among the general populace [20, 21]. Myocarditis is a complex disorder involving multiple immunological mechanisms in its development and progression. The precise etiological factor that initiates its pathogenesis has yet to be unequivocally determined, but a convergence of various endogenous and environmental factors has been demonstrated to incite and perpetuate myocardium-specific autoimmune processes. The immunological characteristics linked to the initiation of myocarditis, its acute phase, and the progressions to DCM have been the subject of extensive research. Several animal models have been widely used to mimic myocarditis in investigations, ranging from an experimental autoimmune myocarditis (EAM) murine model, troponin-induced myocarditis, and coxsackievirus B3 (CVB3)-induced viral myocarditis. However, none of these models could simulate the pathogenesis of human myocarditis.

We used hiPSC-CMs to investigate the pathogenesis of myocardial cell injury caused by inflammation. Autoimmunity triggered by innate and adaptive response after myocardial damage plays a pivotal role in the process of acute and chronic myocarditis. In individuals suffering from acute myocarditis, histological examinations have revealed the aggregation of T cells, alongside other inflammatory cell types, in intimate proximity to the damaged cardiomyocytes [21]. Therefore, we co-cultured hiPSC-CMs with activated CD4 + T or CD8 + T cells, combined with proteomic analysis, to obtain differentially expressed proteins of hiPSC-CMs both before and after T cell treatment. Our results show that both CD4 + T cells and CD8 + T cells can induce an immune response to prevent virus invasion. However, CD4 + T cells damage cardiomyocytes more severely than CD8 + T cells, which may be due to a tendency to produce more interferon and tumor necrosis factor (Fig. 3). A multitude of investigations, predominantly carried out in murine models, have underscored the crucial involvement of CD4 + T cells in the pathogenesis and advancement of myocarditis. Transgenic mice expressing a targeted CD4 + T cell receptor for cardiac myosin exhibit spontaneous myocarditis, which subsequently evolves into fatal dilated cardiomyopathy [22]. Experiments employing knockout mice and adoptive transfer approaches have additionally substantiated the indispensable contribution of CD4 + T cells in the progression of myocarditis, mirroring the characteristics observed in human myocarditis [23]. In a notable investigation employing an experimental model of CVB3-induced myocarditis, the ablation of CD4 + T cells demonstrated a mitigating effect on myocardial inflammation, whereas the sole deletion of CD8 + T cells had no discernible impact on myocarditis-associated inflammation. The concurrent deletion of both CD4 + and CD8 + T cells exhibited the most notable protective effects, underscoring the significance of CD4 + helper T cells in the pathogenesis of myocarditis. Immune responses in myocarditis are closely linked to the production of T cell cytokines, which have been demonstrated in experimental models to govern the progression and outcomes of the disease [7]. Our findings suggest that CD4 cells have a direct cardiotoxic effect, as evidenced by elevated levels of LDH, CK-MB, and cTnI. This is consistent with previous research indicating that CD4 T cell responses play a critical role in driving autoimmune myocarditis. Furthermore, the innate cellular response not only plays a crucial role in initiating autoimmunity but also in sustaining the adaptive T cell-mediated response and facilitating the progression towards chronic myocarditis. Irrespective of the underlying cause, an acute inflammatory response can progress to subacute and chronic stages, ultimately resulting in tissue remodeling, fibrosis, and the deterioration of myocardial architecture and contractile function. Interestingly, we found that the activated CD8 + T cells seriously affected the expression of proteins associated with heart contraction, while CD4 + T cells did not exhibit changes in these proteins. These results suggest that the dysfunction of cardiac contraction and conduction function caused by myocarditis may be mainly caused by the damage that CD8 + T cells does to myocardial cells. Previous studies have reported significant metabolic changes during inflammatory response in the heart and ranging across the generation, transportation, and utilization of energy metabolism [24–26]. Recent surveys have reported that SARS-CoV-2 infection can disrupt energy metabolism, resulting in reduced ATP production and elevated production of reactive oxygen species (ROS) [27, 28]. Our study found that both CD4 + and CD8 + T cells significantly downregulated the expression of proteins associated with lipid metabolism in hiPSC-CMs (Fig. 3B, D). For example, these include fatty acid metabolism, steroid metabolism, and alcohol metabolism processes. In addition, in CD8 + T-treated hiPSC-CMs, we found that protein expression related to glucose metabolism and hormone metabolism, such as that of glucose transport, fructose metabolism, prostaglandin metabolism, and progesterone metabolism, was downregulated. These results were interesting. Recently, a study reported beneficial effects of sodium-dependent glucose transporter 2 (SGLT2) inhibitors in EAM [29]. Indeed, the use of glucose-insulin-potassium (GIK) has been recognized as a beneficial adjunct in the treatment of myocarditis [30]. GIK therapy aims to optimize cardiac metabolism and improve myocardial function. Additionally, patients with sepsis, injury, or critical illness often experience significant hormonal and metabolic alterations. These changes can be profound and complex, requiring appropriate management strategies to support overall patient well-being. Zhang et al. reported that insulin alleviates myocarditis in an experimental autoimmune myocarditis EAM model [31]. Sex hormones play a crucial role in modulating the acute inflammatory response to injury, including myocardial depression and apoptosis. Gender has also been found to impact the inflammatory response and outcomes during acute stimulation [32]. Chen et al. conducted a study and found that corticosteroids can enhance cardiac function in patients with viral myocarditis and low left ventricular ejection fraction [33]. This suggests that corticosteroid treatment may be beneficial in managing viral myocarditis in specific patient populations with impaired cardiac function. These results may indicate that CD8 T cells can cause cardiac systolic dysfunction through metabolic disorder.

Proteomics can accurately demonstrate dynamic changes in protein content in the body during disease, and it was widely used in studies of various cardiovascular diseases. Our study showed significant changes of proteome profilings of hiPSC-CMs after the stimulation with T cells. Moreover, we also discovered that NLRs changed significantly during T cell-mediated cardiomyocyte injury. The NLRs are a group of intracellular proteins that are essential for controlling host innate immune response. NLRs, or Nucleotide-binding oligomerization domain (NOD)-like receptors, are a significant family of intracellular Pattern Recognition Receptors (PRRs). They play a crucial role in detecting and responding to invading pathogens, as well as maintaining normal cellular homeostasis. One notable function of NLRs is their ability to form multi-protein complexes known as inflammasomes. Inflammasomes are activated in response to specific danger signals, such as pathogenic components, damage-associated molecular patterns (DAMPs), or other stress signals. Upon activation, the inflammasome complex triggers a proteolytic cascade, leading to the activation of inflammatory cytokines like IL-1β and IL-18, which are important mediators of the immune response. Additionally, the activation of certain types of inflammasomes can induce a programmed cell death process called apoptosis. The precise mechanisms by which NLRs and inflammasomes regulate immune responses and maintain homeostasis are still being actively studied. However, their involvement in both pathogen defense and immune-mediated inflammation highlights their significance in the immune system’s overall functioning [34, 35]. The research conducted by Liu et al. corroborates the notion that mitochondrial calpain-1 is capable of triggering the NLRP3 inflammasome via ATP5A1 cleavage and eliciting mitochondrial ROS in CVB3-induced myocarditis [36]. Yang et al. showed that sulfur dioxide can attenuate sepsis-induced cardiac dysfunction in rats by inhibiting the activation of the NLRP3 inflammasome [37]. While earlier studies have suggested that NLR signaling plays a crucial role in cardiac injury, there is limited evidence from human samples to support this notion. Further research is needed to explore the potential therapeutic implications of targeting the NLRP3 inflammasome in human myocarditis. Our study demonstrated that CD4 cells act directly to produce cardiomyocyte injury by activating the NLR signal pathway, which could be a potential target for future strategies.

## Conclusions

In this study, we established hiPSC-CMs and co-clutured with T cell to construct the myocardial cell injury caused by inflammation. By using the quantitative proteomics combined with tissue and cell immunofluorescence examination, we established a proteome map of inflammatory myocardia and identified a series of molecular dysfunctions of hiPSC-CMs. The results of our study can elucidate the pathogenesis of T cell-mediated myocardial cell injury and provide guidance in the discovery of improved clinical treatments for myocarditis.

### Electronic supplementary material

Below is the link to the electronic supplementary material.

Supplementary Material 1

Supplementary Material 2

Supplementary Material 3

Supplementary Material 4

Supplementary Material 5

## Data Availability

All proteomics data generated in this study have been deposited to ProteomeXchange Consortium via the iProX [38, 39] partner repository with the identifier PXD039790.

## Abbreviations

AGC: Automatic gain control
CVB3: Coxsackievirus B3
cTnI: Cardiac troponin I
cTnT: Cardiac troponin T
CK-MB: Creatine kinase isoenzyme MB
DCM: Dilated cardiomyopathy
DDA: Data-dependent acquisition
DIA: Data-independent acquisition
EAM: Experimental autoimmune myocarditis
ELISA: Enzyme-linked immunosorbent assay
H&E: Hematoxylin and eosin
hiPSCs: Human induced pluripotent stem cells
hiPSC-CMs: Human induced pluripotent stem cell-derived cardiomyocytes
LDH: Lactate dehydrogenase
MS: Mass spectrometry
NCE: Normalized collision energy
PBMCs: Peripheral blood mononuclear cells
PCoA: Principal coordinates analysis

## References

[1] Cooper LT. Jr. Myocarditis. N Engl J Med. 2009;360(15):1526–38. 19357408 10.1056/NEJMra0800028 PMC5814110

[2] Tschöpe C, Ammirati E, Bozkurt B, Caforio ALP, Cooper LT, Felix SB, et al. Myocarditis and inflammatory cardiomyopathy: current evidence and future directions. Nat Rev Cardiol. 2021;18(3):169–93. 33046850 10.1038/s41569-020-00435-x PMC7548534

[3] Heymans S, Lakdawala NK, Tschöpe C, Klingel K. Dilated cardiomyopathy: causes, mechanisms, and current and future treatment approaches. Lancet. 2023;402(10406):998–1011. 37716772 10.1016/S0140-6736(23)01241-2

[4] Maron BJ, Udelson JE, Bonow RO, Nishimura RA, Ackerman MJ, Estes NAM 3, et al. Eligibility and disqualification recommendations for competitive athletes with Cardiovascular abnormalities: Task Force 3: hypertrophic cardiomyopathy, Arrhythmogenic Right Ventricular Cardiomyopathy and other Cardiomyopathies, and Myocarditis: A Scientific Statement from the American Heart Association and American College of Cardiology. J Am Coll Cardiol. 2015;66(21):2362–71. 26542657 10.1016/j.jacc.2015.09.035

[5] Golpour A, Patriki D, Hanson PJ, McManus B, Heidecker B. Epidemiological Impact of Myocarditis. J Clin Med. 2021;10(4):603. 33562759 10.3390/jcm10040603 PMC7915005

[6] Kaya Z, Katus HA, Rose NR. Cardiac troponins and autoimmunity: their role in the pathogenesis of myocarditis and of heart failure. Clin Immunol. 2010;134(1):80–8. 19446498 10.1016/j.clim.2009.04.008 PMC2819185

[7] Stephenson E, Savvatis K, Mohiddin SA, Marelli-Berg FM. T-cell immunity in myocardial inflammation: pathogenic role and therapeutic manipulation. Br J Pharmacol. 2017;174(22):3914–25. 27590129 10.1111/bph.13613 PMC5659997

[8] Fairweather D, Kaya Z, Shellam GR, Lawson CM, Rose NR. From infection to autoimmunity. J Autoimmun. 2001;16(3):175–86. 11334481 10.1006/jaut.2000.0492

[9] Lv H, Havari E, Pinto S, Gottumukkala RV, Cornivelli L, Raddassi K, et al. Impaired thymic tolerance to α-myosin directs autoimmunity to the heart in mice and humans. J Clin Invest. 2011;121(4):1561–73. 21436590 10.1172/JCI44583 PMC3069776

[10] Lichtman AH. The heart of the matter: protection of the myocardium from T cells. J Autoimmun. 2013;45:90–6. 23810579 10.1016/j.jaut.2013.05.004 PMC3783609

[11] Błyszczuk P. Myocarditis in humans and in Experimental Animal models. Front Cardiovasc Med. 2019;6:64. 31157241 10.3389/fcvm.2019.00064 PMC6532015

[12] Takahashi K, Tanabe K, Ohnuki M, Narita M, Ichisaka T, Tomoda K, et al. Induction of pluripotent stem cells from adult human fibroblasts by defined factors. Cell. 2007;131(5):861–72. 18035408 10.1016/j.cell.2007.11.019

[13] Yu J, Vodyanik MA, Smuga-Otto K, Antosiewicz-Bourget J, Frane JL, Tian S, et al. Induced pluripotent stem cell lines derived from human somatic cells. Science. 2007;318(5858):1917–20. 18029452 10.1126/science.1151526

[14] Smith AS, Macadangdang J, Leung W, Laflamme MA, Kim DH. Human iPSC-derived cardiomyocytes and tissue engineering strategies for disease modeling and drug screening. Biotechnol Adv. 2017;35(1):77–94. 28007615 10.1016/j.biotechadv.2016.12.002 PMC5237393

[15] Tang X, Liu H, Rao R, Huang Y, Dong M, Xu M, et al. Modeling drug-induced mitochondrial toxicity with human primary cardiomyocytes. Sci China Life Sci. 2024;67(2):301–19. 37864082 10.1007/s11427-023-2369-3

[16] Huang W, Zhou R, Jiang C, Wang J, Zhou Y, Xu X, et al. Mitochondrial dysfunction is associated with hypertrophic cardiomyopathy in pompe disease-specific induced pluripotent stem cell-derived cardiomyocytes. Cell Prolif. 2024;57(4):e13573. 37916452 10.1111/cpr.13573 PMC10984102

[17] Chen P, Xiao Y, Wang Y, Zheng Z, Chen L, Yang X, et al. Intracellular calcium current disorder and disease phenotype in OBSCN mutant iPSC-based cardiomyocytes in arrhythmogenic right ventricular cardiomyopathy. Theranostics. 2020;10(24):11215–29. 33042279 10.7150/thno.45172 PMC7532677

[18] Xiong Y, Wang Y, Zhang J, Zhao N, Zhang H, Zhang A, et al. hPMSCs protects against D-galactose-induced oxidative damage of CD4(+) T cells through activating akt-mediated Nrf2 antioxidant signaling. Stem Cell Res Ther. 2020;11(1):468. 33148324 10.1186/s13287-020-01993-0 PMC7641865

[19] Banerjee SK, Chatterjee A, Gupta S, Nagar A. Activation and regulation of NLRP3 by sterile and infectious insults. Front Immunol. 2022;13:896353. 35663964 10.3389/fimmu.2022.896353 PMC9161712

[20] Caforio AL, Pankuweit S, Arbustini E, Basso C, Gimeno-Blanes J, Felix SB, et al. Current state of knowledge on aetiology, diagnosis, management, and therapy of myocarditis: a position statement of the European Society of Cardiology Working Group on Myocardial and Pericardial diseases. Eur Heart J. 2013;34(33):2636–48. 2648a-2648d. 23824828 10.1093/eurheartj/eht210

[21] Fabre A, Sheppard MN. Sudden adult death syndrome and other non-ischaemic causes of sudden cardiac death. Heart. 2006;92(3):316–20. 15923280 10.1136/hrt.2004.045518 PMC1860827

[22] Nindl V, Maier R, Ratering D, De Giuli R, Züst R, Thiel V, et al. Cooperation of Th1 and Th17 cells determines transition from autoimmune myocarditis to dilated cardiomyopathy. Eur J Immunol. 2012;42(9):2311–21. 22730043 10.1002/eji.201142209

[23] Afanasyeva M, Georgakopoulos D, Fairweather D, Caturegli P, Kass DA, Rose NR. Novel model of constrictive pericarditis associated with autoimmune heart disease in interferon-gamma-knockout mice. Circulation. 2004;110(18):2910–7. 15505106 10.1161/01.CIR.0000147538.92263.3A

[24] Ebermann L, Wika S, Klumpe I, Hammer E, Klingel K, Lassner D, et al. The mitochondrial respiratory chain has a critical role in the antiviral process in Coxsackievirus B3-induced myocarditis. Lab Invest. 2012;92(1):125–34. 21968812 10.1038/labinvest.2011.145

[25] Remels AHV, Derks WJA, Cillero-Pastor B, Verhees KJP, Kelders MC, Heggermont W, et al. NF-κB-mediated metabolic remodelling in the inflamed heart in acute viral myocarditis. Biochim Biophys Acta Mol Basis Dis. 2018;1864(8):2579–89. 29730342 10.1016/j.bbadis.2018.04.022

[26] Zhu H, Galdos FX, Lee D, Waliany S, Huang YV, Ryan J, et al. Identification of pathogenic Immune Cell subsets Associated with checkpoint inhibitor-Induced myocarditis. Circulation. 2022;146(4):316–35. 35762356 10.1161/CIRCULATIONAHA.121.056730 PMC9397491

[27] Ho HT, Peischard S, Strutz-Seebohm N, Klingel K, Seebohm G. Myocardial damage by SARS-CoV-2: emerging mechanisms and therapies. Viruses. 2021;13(9):1880. 34578462 10.3390/v13091880 PMC8473126

[28] Leng L, Ma J, Zhang PP, Xu SC, Li X, Jin Y, et al. Spatial region-resolved proteome map reveals mechanism of COVID-19-associated heart injury. Cell Rep. 2022;39(11):110955. 35679865 10.1016/j.celrep.2022.110955 PMC9135696

[29] Long Q, Li L, Yang H, Lu Y, Yang H, Zhu Y, et al. SGLT2 inhibitor, canagliflozin, ameliorates cardiac inflammation in experimental autoimmune myocarditis. Int Immunopharmacol. 2022;110:109024. 35841866 10.1016/j.intimp.2022.109024

[30] Tessier JP, Thurner B, Jüngling E, Lückhoff A, Fischer Y. Impairment of glucose metabolism in hearts from rats treated with endotoxin. Cardiovasc Res. 2003;60(1):119–30. 14522413 10.1016/S0008-6363(03)00320-1

[31] Zhang Y, Zhuang R, Geng C, Cai X, Lei W, Tian N, et al. Insulin promotes T cell recovery in a murine model of autoimmune myocarditis. Clin Exp Immunol. 2013;171(1):46–53. 23199322 10.1111/j.1365-2249.2012.04662.x PMC3530094

[32] Meldrum DR, Wang M, Tsai BM, Kher A, Pitcher JM, Brown JW, et al. Intracellular signaling mechanisms of sex hormones in acute myocardial inflammation and injury. Front Biosci. 2005;10:1835–67. 15769671 10.2741/1665

[33] Chen HS, Wang W, Wu SN, Liu JP. Corticosteroids for viral myocarditis. Cochrane Database Syst Rev. 2013; 2013(10):CD004471. 10.1002/14651858.CD004471.pub3 PMC8094275 24136037

[34] Liu P, Lu Z, Liu L, Li R, Liang Z, Shen M, et al. NOD-like receptor signaling in inflammation-associated cancers: from functions to targeted therapies. Phytomedicine. 2019;64:152925. 31465982 10.1016/j.phymed.2019.152925

[35] Zheng S, Que X, Wang S, Zhou Q, Xing X, Chen L, et al. ZDHHC5-mediated NLRP3 palmitoylation promotes NLRP3-NEK7 interaction and inflammasome activation. Mol Cell. 2023;83(24):4570–e45857. 38092000 10.1016/j.molcel.2023.11.015

[36] Liu X, Li M, Chen Z, Yu Y, Shi H, Yu Y, et al. Mitochondrial calpain-1 activates NLRP3 inflammasome by cleaving ATP5A1 and inducing mitochondrial ROS in CVB3-induced myocarditis. Basic Res Cardiol. 2022;117(1):40. 35997820 10.1007/s00395-022-00948-1 PMC9399059

[37] Yang L, Zhang H, Chen P. Sulfur dioxide attenuates sepsis-induced cardiac dysfunction via inhibition of NLRP3 inflammasome activation in rats. Nitric Oxide. 2018;81:11–20. 30273666 10.1016/j.niox.2018.09.005

[38] Ma J, Chen T, Wu S, Yang C, Bai M, Shu K, et al. iProX: an integrated proteome resource. Nucleic Acids Res. 2019;47(D1):D1211–7. 30252093 10.1093/nar/gky869 PMC6323926

[39] Chen T, Ma J, Liu Y, Chen Z, Xiao N, Lu Y, et al. iProX in 2021: connecting proteomics data sharing with big data. Nucleic Acids Res. 2022;50(D1):D1522–7. 34871441 10.1093/nar/gkab1081 PMC8728291

