# Supplementary material for: Effect and mechanism of T lymphocytes on human induced pluripotent stem cell-derived cardiomyocytes via Proteomics
Source: Stem Cell Res Ther. 2024 Jul 29;15:236. doi: 10.1186/s13287-024-03791-4 (PMC11288085; doi:10.1186/s13287-024-03791-4)
Supplement: Supplementary file 5 — Supplementary Material 5 [file 13287_2024_3791_MOESM5_ESM.pdf]

Supplementary Figures

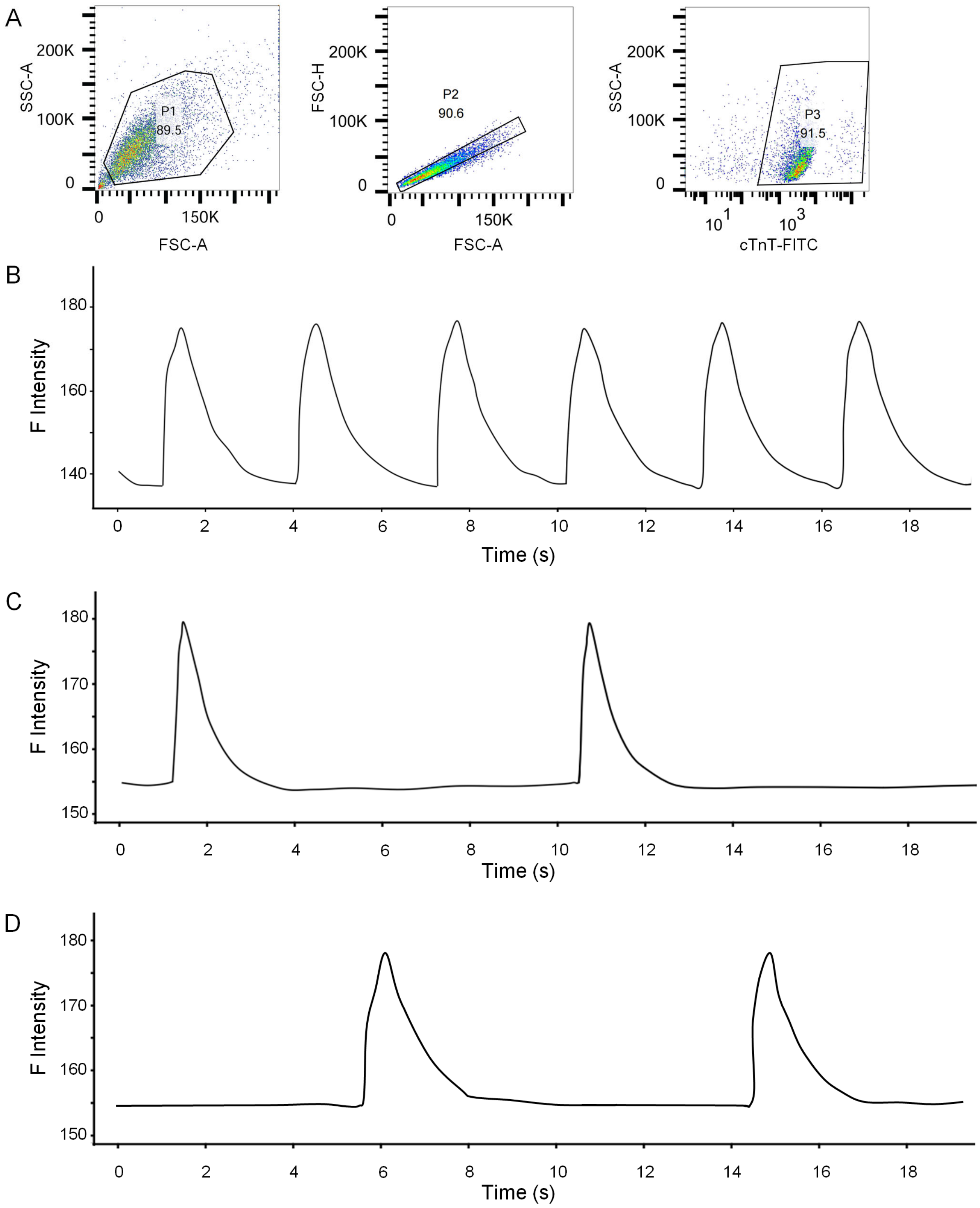

Figure S1. Evaluation of the purity and beating property of human induced pluripotent stem cell-derived cardiomyocytes (hiPSC-CMs). (A) Representative plots of flow cytometry showing the cTnT expression in iPSC-CMs. The purity of hiPSC-CMs is about 91.5% after selection by the method of flow cytometry. Representative calcium transient recordings of normal hiPSC-CMs (B) and hiPSC-CMs co- cultured with the activated CD4+ T cells (C) and CD8+ T cells (D). After co-cultured with the CD4+ and CD8+ T cells, the beat rates of hiPSC-CMs decreased.

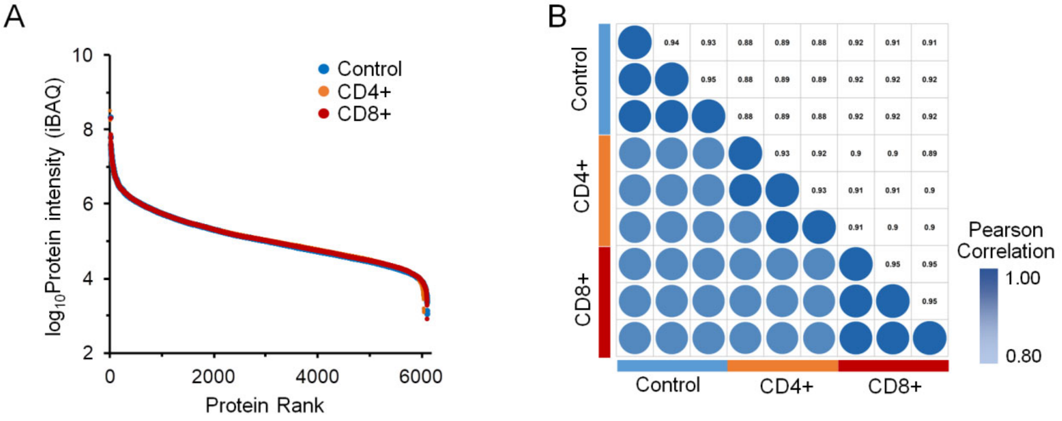

Figure S2. Proteome profile of normal hiPSC-CMs and hiPSC-CMs co-cultured with activated T cells. (A) The protein intensity distributions of hiPSC-CMs with different inflammatory T cells infiltration. (B) Correlation analysis of proteomic profiles of hiPSC-CMs in the control, CD4+T cell, and CD8+T cell treated groups. The pairwise Pearson's correlation coefficients of hypoxia and control samples were calculated by the pattern of relative protein abundance (range: 0.80–1.00).
